# Acute Ischemic Stroke and Rapid Resolution of a Large Left Ventricular Thrombus With Low-Molecular-Weight Heparin: A Case Report

**DOI:** 10.7759/cureus.95738

**Published:** 2025-10-30

**Authors:** Mohammed H Elaghory, Mohammad Bayer, Bilal Nasir, Pagadala Sridhar

**Affiliations:** 1 Stroke/Cardiology, Hywel Dda University Health Board-Glangwili General Hospital, Carmarthen, GBR; 2 General Medicine, Hywel Dda University Health Board-Glangwili General Hospital, Carmarthen, GBR; 3 Stroke Medicine, Hywel Dda University Health Board-Glangwili General Hospital, Carmarthen, GBR; 4 Medicine, Hywel Dda University Health Board-Glangwili General Hospital, Carmarthen, GBR

**Keywords:** echocardiography, hypercoagulable state, ischemic stroke, left ventricular thrombus, low-molecular-weight heparin

## Abstract

We present the case of a 62-year-old male who presented with an acute ischemic stroke and was subsequently diagnosed with a large mobile left ventricular (LV) thrombus. Although surgical intervention was initially considered, the cardiothoracic team opted for conservative management. The patient was managed conservatively with aspirin and later therapeutic low-molecular-weight heparin (LMWH) due to the LV thrombus. Notably, follow-up echocardiography after two weeks revealed complete resolution of the LV thrombus, an outcome that highlights the efficacy of LMWH and the potential for rapid resolution without surgical intervention. The patient experienced further complications, including a new cerebellar infarct and venous thromboembolism, prompting investigation for underlying hypercoagulable states such as lupus antibodies. This case underscores the critical importance of early investigation for secondary causes of ischemic stroke and demonstrates the significant therapeutic potential of LMWH in achieving rapid LV thrombus resolution, thereby preventing the need for more invasive procedures.

## Introduction

Ischemic stroke remains one of the leading causes of morbidity and mortality worldwide and requires prompt identification and management of the underlying aetiology to prevent recurrence [1]. Cardioembolic stroke, particularly secondary to left ventricular thrombus (LVT), constitutes an important subset with a high risk of recurrent embolic events [2]. LVT most often develops after acute myocardial infarction (AMI) with severe left ventricular dysfunction but may also arise in non-ischemic cardiomyopathies [3]. Current guidelines recommend anticoagulation traditionally with vitamin K antagonists (VKAs), although direct oral anticoagulants (DOACs) are increasingly being explored [2,4]. The optimal timing and choice of anticoagulation, particularly following acute ischemic stroke, remain areas of ongoing clinical debate [5,6].

This case report describes an unusual presentation of a large (5.4 cm) mobile LVT in a patient with only mildly reduced left ventricular ejection fraction (45-49%), an uncommon association. It further highlights the complete resolution of the thrombus within two weeks of LMWH (Clexane) therapy, emphasizing the importance of early etiological investigation and demonstrating the potential role of LMWH as an effective treatment option when oral anticoagulation is unsuitable.

## Case presentation

**Figure 1:**
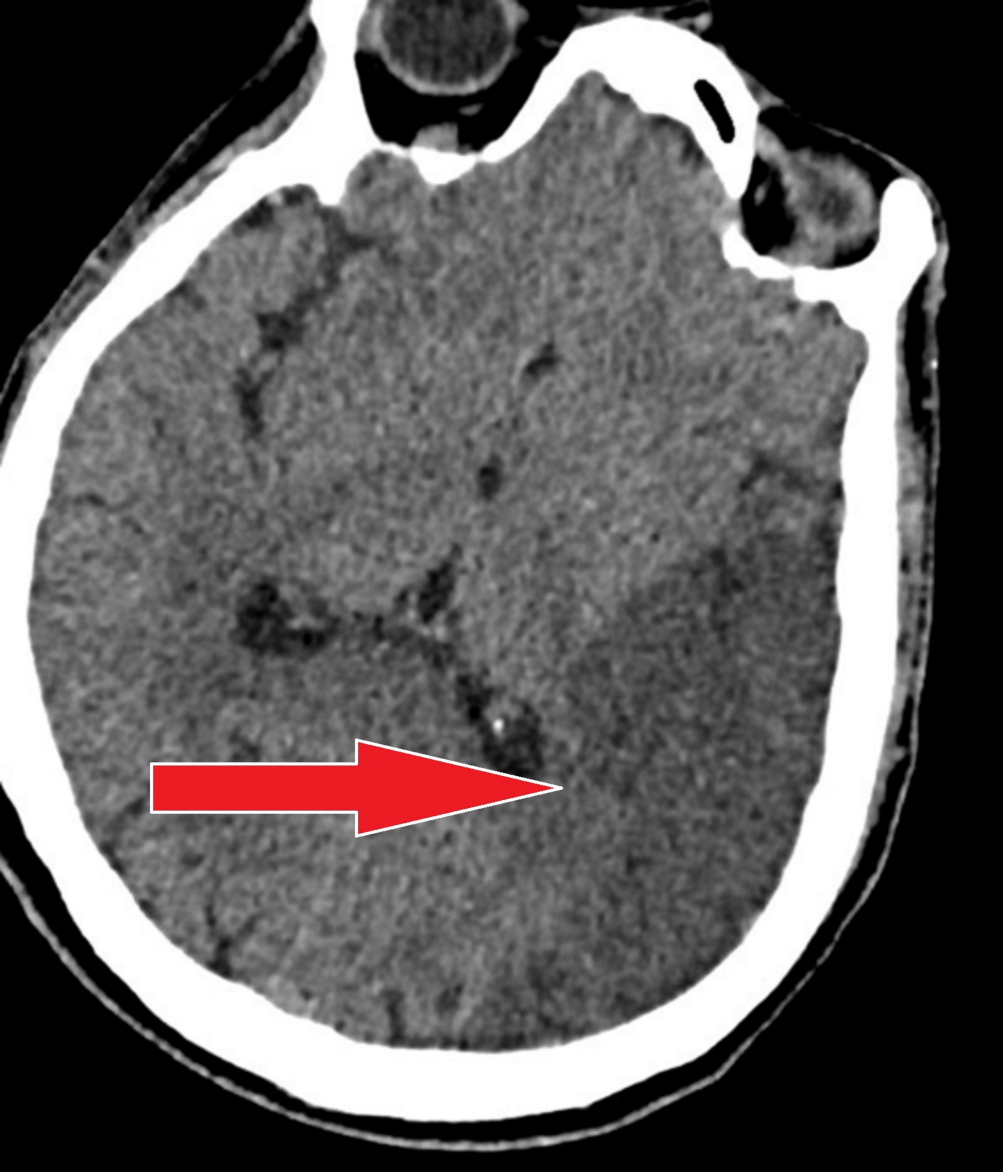
Non-contrast CT head scan showing acute and established transcortical infarcts (red arrow) in the left posterior parietal and temporal lobes. This finding confirmed an ischemic stroke involving the left middle cerebral artery (MCA) territory and guided further vascular imaging and management decisions.

A 62-year-old male, with no significant past medical history, presented with acute confusion that began one day prior to admission. On presentation, his National Institutes of Health Stroke Scale (NIHSS) score was 6, characterized by slurred speech, movement in all four limbs, and a Glasgow Coma Scale (GCS) of 13. Initial investigations included an electrocardiogram (ECG), which showed widespread T-wave inversions and sinus rhythm. A computed tomography (CT) head scan revealed acute and established transcortical infarcts in the left posterior parietal and temporal lobes (see Figure 1). CT angiography demonstrated significant stenosis or partial thrombus in the distal M2 segment of the left middle cerebral artery (MCA).

The case was discussed with the neuroradiology intervention team; however, mechanical thrombectomy was deemed unsuitable as imaging confirmed an already established infarction in the affected area. The patient was initiated on an aspirin 300 mg loading dose, consistent with standard stroke management guidelines, and subsequently maintained on antiplatelet therapy. A speech and language therapy (SALT) assessment confirmed he passed the swallowing test.

**Figure 2:**
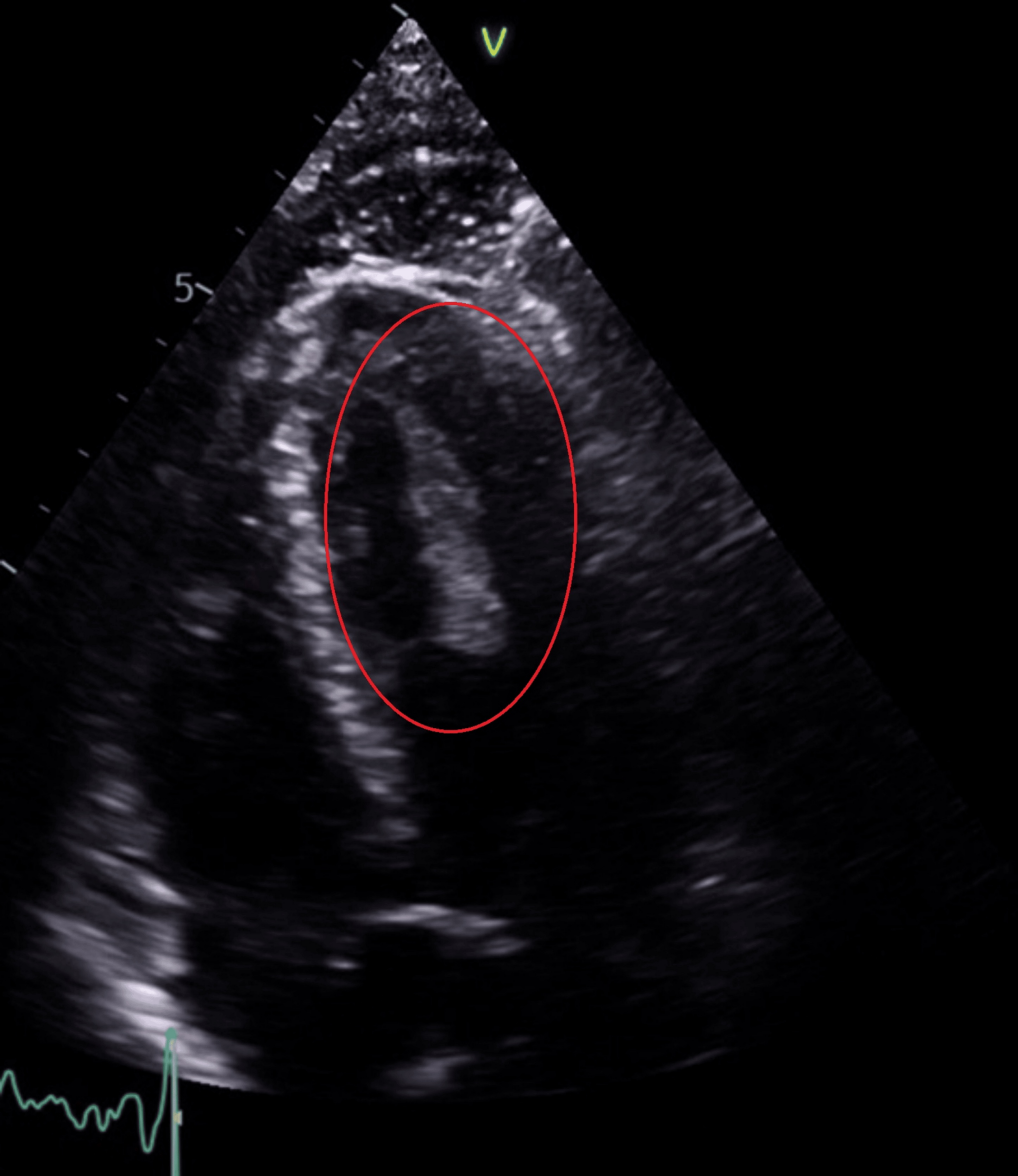
Transthoracic echocardiogram (apical five-chamber view) demonstrating a large, mobile left ventricular (LV) thrombus (red circle) measuring approximately 5.4 × 1.1 cm. The thrombus was attached near the apical region with mild LV systolic dysfunction (estimated EF 45–49%), representing a potential cardioembolic source.

On the second day of admission, an echocardiogram was performed, which unexpectedly revealed a large mobile LV thrombus (5.4 cm × 1.1 cm) and mild left ventricular systolic dysfunction (LVEF 45-49%, visually estimated) (Figure 2, Video 1). Following this critical finding, therapeutic LMWH (enoxaparin 80 mg subcutaneously twice daily) was initiated. Consultation with the cardiothoracic team advised continuation of anticoagulation and recommended a bubble contrast echocardiogram, which could not be performed due to persistent confusion. A CT thorax-abdomen-pelvis (CT TAP) was performed to characterize the LV mass and exclude other complications, revealing multiple renal infarcts but no additional abnormalities. Kidney, liver, and hematologic profiles, including coagulation studies, remained within normal limits throughout admission. 

**Video 1:** Transthoracic echocardiogram demonstrating a large, mobile left ventricular thrombus (5.4 cm × 1.1 cm) attached to the apical region.

**Figure 3:**
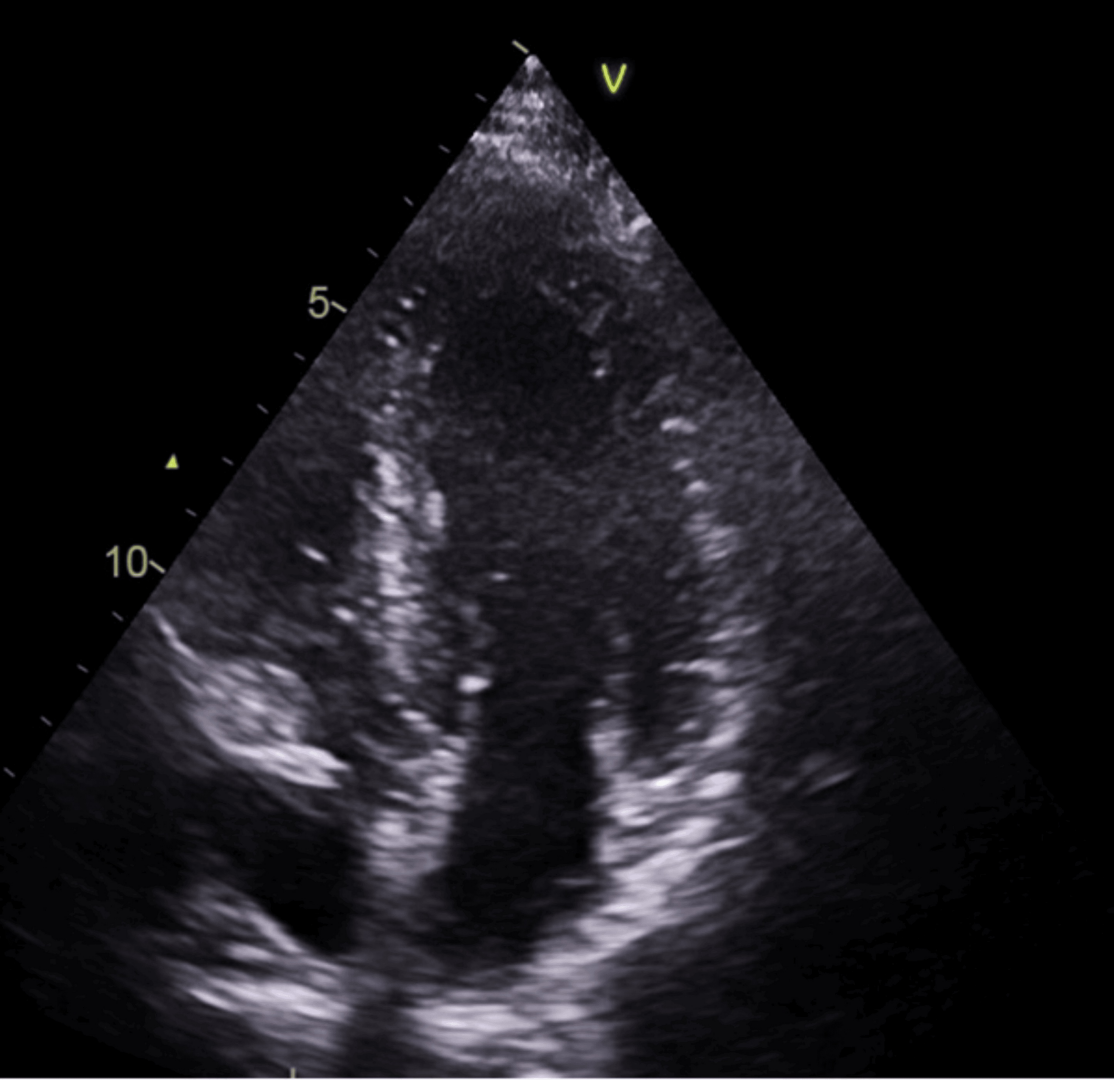
Follow-up transthoracic echocardiogram (apical four-chamber view) showing complete resolution of the previously identified LV thrombus. This image, obtained two weeks after initiation of therapeutic low-molecular-weight heparin (Clexane 1 mg/kg BID), demonstrates rapid thrombus resolution and supports the efficacy of LMWH in acute management.

On day 3, the patient developed a seizure, prompting a repeat CT head scan, which demonstrated a new right cerebellar infarct. Doppler ultrasound of the lower limbs confirmed venous thromboembolism (VTE) in the left leg. He was started on levetiracetam 250 mg twice daily, later increased to 500 mg twice daily for seizure control. A repeat echocardiogram performed after two weeks demonstrated complete resolution of the LV thrombus (Figure 3, Video 2). Investigations for underlying prothrombotic conditions, including lupus antibodies, were negative.

**Video 2:** Transthoracic echocardiogram showing complete resolution of left ventricular thrombus after two weeks of low-molecular-weight heparin (LMWH) therapy.

The patient’s left ventricular function remained stable (LVEF 45-49%) with an akinetic apex. Beta-blockers were not initiated as his heart rate remained around 60 bpm. He was started on low-dose ACE inhibitor (Ramipril 1.25 mg daily) and dapagliflozin for cardioprotective management. The patient remained clinically stable with no further seizures, although confusion persisted at discharge.

Table 1 shows the summary of key clinical events and management.

**Table 1:** Summary of key clinical events and management NIHSS: National Institutes of Health Stroke Scale, CT: computed tomography, CTA: computed tomography angiography, SALT: speech and language therapy, LV: left ventricular, LMWH: low-molecular-weight heparin, VTE: venous thromboembolism

Day	Event	Investigation/findings	Management
1	Presentation with acute confusion, NIHSS 6	CT head: acute + established infarcts; CTA: distal M2 thrombus	Aspirin 300 mg started; SALT passed
2	Cardiac evaluation	Echocardiogram: large mobile LV thrombus (5.4 × 1.1 cm), LVEF 45–49%	Started on therapeutic LMWH (enoxaparin 80 mg BID)
3	Seizure episode	CT head: new right cerebellar infarct	Levetiracetam initiated; continued LMWH
4–14	Monitoring	Stable renal/liver function, confirmed VTE	Continued LMWH, supportive care
14	Re-evaluation	Echocardiogram: complete thrombus resolution	Continued secondary stroke prevention, added Ramipril and dapagliflozin

## Discussion

This case highlights several important clinical considerations in the management of acute ischemic stroke, particularly when complicated by LV thrombus. The initial presentation with acute stroke, followed by the incidental discovery of a large mobile LV thrombus, underscores the necessity of a thorough diagnostic workup for secondary stroke aetiologies [1]. While the patient's initial stroke was attributed to M2 segment stenosis or thrombus, the presence of a mobile LV thrombus immediately raised concerns for a cardioembolic source, especially given the subsequent new cerebellar infarct and VTE, suggesting a systemic prothrombotic state [2].

The rapid and complete resolution of the large mobile LV thrombus within two weeks of initiating therapeutic LMWH is a notable finding. Previous studies have shown that LMWH can be effective in treating LV thrombi, with some reporting high rates of resolution [5]. For instance, Meurin et al. (2005) demonstrated that enoxaparin was well tolerated and efficient in achieving LV thrombus disappearance or reduction, with 73% resolution in their cohort [5]. While VKAs have traditionally been the mainstay for LV thrombus treatment, DOACs are emerging as alternatives with comparable safety profiles and potentially higher resolution rates in some studies [7,8].

The timing of anticoagulation initiation after ischemic stroke remains critical, as it requires balancing the risk of recurrent embolic events against the potential for haemorrhagic transformation [4,9]. In this case, LMWH was initiated promptly following echocardiographic confirmation of a large mobile thrombus, leading to rapid thrombus resolution without evidence of haemorrhagic complications. Recent evidence continues to explore the relative efficacy of LMWH, VKAs, and DOACs in LV thrombus management. A 2023 meta-analysis reported similar rates of thrombus resolution between DOACs and VKAs, with a potentially lower bleeding risk for DOACs, although LMWH remains a valuable early treatment option in acute or unstable cases, particularly when oral therapy is unsuitable [8]. However, causality between LMWH therapy and thrombus resolution cannot be definitively established from a single case, and further prospective data are warranted.

The development of a new cerebellar infarct and VTE despite anticoagulation, along with persistent confusion, points toward a possible underlying hypercoagulable disorder. The ongoing investigation for lupus antibodies is pertinent, as antiphospholipid syndrome (APS) is a known cause of recurrent thrombotic events, including stroke and VTE, and can be associated with LV thrombus [10]. Management of stroke in the context of APS often involves long-term anticoagulation, typically with warfarin, although the optimal intensity and role of DOACs remain debated [10].

## Conclusions

This case highlights the complex interplay between acute ischemic stroke, LV thrombus, and potential underlying hypercoagulable states. The rapid and complete resolution of a large mobile LV thrombus with therapeutic LMWH underscores its potential role in acute management, particularly when oral anticoagulation is unsuitable or contraindicated. While the outcome in this single case was favourable, these findings should be interpreted with caution. This report reinforces the importance of comprehensive and timely diagnostic evaluation for secondary causes of ischemic stroke, including echocardiography for LV thrombus detection, and investigation for prothrombotic conditions in cases of recurrent or atypical thrombotic events. Further studies are warranted to establish optimal anticoagulation strategies, timing, and duration for LV thrombus management in the setting of acute ischemic stroke.
